# Hexane Extract of *Chloranthus japonicus* Increases Adipocyte Differentiation by Acting on Wnt/β-Catenin Signaling Pathway

**DOI:** 10.3390/life11030241

**Published:** 2021-03-15

**Authors:** Ui Jeong Yun, Chu Won Nho, Kye Won Park, Dong Kwon Yang

**Affiliations:** 1 Department of Food Science and Biotechnology, Food Clinical Research Center, Sungkyunkwan University, Suwon 16419, Korea; yunc@skku.edu; 2 Smart Farm Research Center, KIST Gangneung Institute of Natural Products, Gangneung, Gangwon-do 25451, Korea; cwnho@kist.re.kr; 3 Department of Veterinary Pharmacology and Toxicology, College of Veterinary Medicine, Chonbuk National University, Iksan, Jeollabuk-do 54596, Korea

**Keywords:** *Chloranthus japonicas*, PPARγ, adipocyte, obesity, metabolism

## Abstract

*Chloranthus japonicus* has been heavily investigated for the treatment of various diseases. This paper attempts to show that *Chloranthus japonicus* can modulate adipocyte differentiation of preadipocytes. To establish this, we investigated the effects of *Chloranthus japonicus* extract in peroxisome proliferator-activated receptor γ (PPARγ) expression, adipogenesis, and the underlying molecular mechanisms in C3H10T1/2 and 3T3-L1 cells. Our data showed that *Chloranthus japonicus* methanol extract increased lipid accumulation and promoted adipocyte differentiation. Further studies on the fractionation with various solvents led to the identification of *Chloranthus japonicus* hexane extract (CJHE) as the most potent inducer of adipocyte differentiation. CJHE consistently increased lipid accumulation and adipocyte marker expression including Pparγ and it acted during the early stages of adipocyte differentiation. Mechanistic studies revealed that CJHE and a Wnt inhibitor similarly stimulated adipogenesis and were active in Wnt-selective reporter assays. The effects of CJHE were inhibited by Wnt3a protein treatment and were significantly blunted in β-catenin-silenced cells, further suggesting that CJHE acted on Wnt pathways to promote adipogenesis. We also showed that *Chloranthus japonicus* extracts generated from different plant parts similarly promoted adipocyte differentiation. These results identified *Chloranthus japonicus* as a pro-adipogenic natural product and suggest its potential use in metabolic syndrome.

## 1. Introduction

Peroxisome proliferator-activated receptor γ (PPARγ), a nuclear hormone receptor, is the master regulator in adipocytes since it is critical for the differentiation of precursor cells into adipocytes [1,2,3,4,5]. Pparγ regulates inflammation and lipid metabolism [1,6,7]. Upon stimulation by fatty acids, prostaglandin J2, or synthetic thiazolidinediones, Pparγ heterodimerized with RXR increased the transcription of C/EBP-α, FABP4, CD36, adiponectin, and LPL [7,8]. 

Besides the effects in adipogenesis, Pparγ modulates systemic glucose metabolism, as shown by the application of thiazolidinediones (TZDs), PPARγ agonists in diabetic mice [1,8]. Although TZDs are used in the diabetes, adverse effects have limited their clinical use [9,10]. However, studies on Pparγ expression and its modification have brought attention back to Pparγ [11,12,13]. The induction of Pparγ expression by the small molecule harmine improves insulin sensitivity with little effect on weight gain, suggesting that inducing Pparγ expression can be used against obesity related insulin dysfunctions [14]. Phosphorylation at S273 of Pparγ is positively associated with obesity in rodents [13]. Furthermore, inhibition of S273 Pparγ phosphorylation ameliorates insulin activity without effects on weight gain and fluid retention [13]. Therefore, the modulation of Pparγ activity and expression can provide alternative therapeutic strategies against insulin resistance and diabetes.

The bioactive compounds in various natural products have been used to treat cancer, inflammation, and obesity [15]. *Chloranthus japonicus* is traditionally used for the treatment of various conditions such as rheumatic arthralgia, bone fractures, pulmonary tuberculosis, and neurasthenia [16,17]. Sesquiterpenoids isolated from *Chloranthus japonicus* exhibit cytotoxic [18] and anti-HIV effects, anti-inflammatory activity [19], and reduce hepatic lipid content [20]. Therefore, we hypothesized that *Chloranthus japonicus* could also modulate adipocyte differentiation in preadipocytes. Here, we showed that *Chloranthus japonicus* extract increased PPARγ expression and adipogenesis by acting on the Wnt pathway.

## 2. Materials and Methods

### 2.1. Chloranthus Japonicus Extraction 

*Chloranthus japonicus* extract was purchased from the Korea Plant Extract Bank and the plants were also collected from May–June from Baek-duk Mountain in Kangwon, Korea. The whole plants, leaves, and stems were dried and powdered, and 23.8 g of the whole plants was extracted with 20 volumes of 70% methanol (400 mL) for 48 hours. The extracts were filtered, concentrated, and freeze-dried as described previously [21]. The extract was fractionated in various solvents of n-hexane, dichloromethane, ethyl acetate, butanol, and water. The extracts were reconstituted in dimethyl sulfoxide (DMSO) at a concentration of 10 mg/mL.

### 2.2. Cell Culture and Adipocyte Differentiation

C3H10T1/2 cells and 3T3-L1 cells were cultured and differentiated as previously described [22]. During differentiation, the media was changed to media of DMEM, 10% FBS, and 5 μg/mL insulin every 2 days. The PPARγ agonist GW1929 was bought from Sigma (St. Louis, MO, USA) and supplemented for C3H10T1/2 differentiation. The differentiated adipocytes were fixed and stained with Oil Red O (Sigma) as described previously [22].

### 2.3. mRNA Expression Analysis

RNA isolated from cells using TRIzol reagent (Invitrogen, Carlsbad, CA, USA). cDNA was synthesized from the total RNA by the AMV Reverse Transcription System kit (Promega, Madison, WI, USA). The gene was amplified in a Thermal Cycler Dice (Takara, Shiga, Japan). Expression was normalized to the levels of acidic ribosomal phosphoprotein P0 (36B4) and the expression level was calculated using 2^−∆CT^ method. The oligonucleotide primer was synthesized from Integrated DNA Technologies (San Diego, CA, USA). The sequences for PCR primers were previously shown [22].

### 2.4. Wnt Activation and Inhibition Studies

Purified Wnt3a protein (R&D Systems, Minneapolis, MN, USA) and Wnt-3a conditioned media was used as previously described [23]. The Wnt inhibitor hexachlorophene (HCP) was purchased from Sigma. The cells were treated with 5 µM HCP, extracts, Wnt conditioned media, or Wnt3a protein and differentiated into adipocytes. For the knock-down studies, β-Catenin siRNA (sc-29210, and control nonspecific siRNA (sc-37007) were purchased from Santa Cruz Biotechnology (Santa Cruz, CA, USA) and were transfected with 50 nmol of siRNA using RNAiMAX (Invitrogen). For the reporter assay, a TOP luciferase vector was co-transfected with renilla into 293T cells using Lipofectamine 2000 (Invitrogen) and HCP or hexane extract was treated for 24 hours. After 48 hours, reporter activity was measured using the Dual-luciferase Reporter Assay System (Promega). 

### 2.5. Statistical Analysis

The data are were analyzed using two-tailed unpaired Student’s *t*-tests. A *p*-value of < 0.05 was used to define statistical significance. 

## 3. Results

### 3.1. Chloranthus Japonicus Methanol Extract Promotes Adipogenesis in C3H10T1/2 and 3T3-L1 Cells

**Figure 1:**
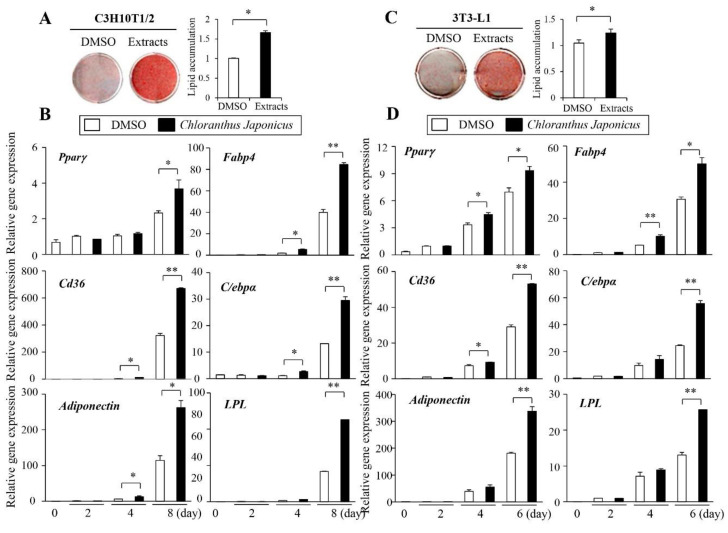
*Chloranthus japonicus* extract promotes the adipocyte differentiation. (**A**) *Chloranthus japonicus* methanol extract increased lipid accumulation in C3H10T1/2 cells (left). Lipid accumulation was determined by Oil Red O and quantified (right). (**B**) *Chloranthus japonicus* methanol extract increased the expression of adipocyte markers. C3H10T1/2 cells were stimulated into adipocytes and treated with *Chloranthus japonicus* methanol extract for the indicated time. The gene expression was measured by real-time PCR. (**C**) *Chloranthus japonicus* methanol extract increased lipid accumulation during the adipocyte differentiation in 3T3-L1 cells (left). Lipid accumulation was quantified (right). (**D**) *Chloranthus japonicus* methanol extract increased the expression of adipogenic genes. 3T3-L1 cells were differentiated and treated with *Chloranthus japonicus* methanol extract for the indicated time. The gene expression levels were measured. The data represent the mean ± standard error of the mean (SEM) of triplicates. Statistical significance was determined relative to the controls by the Student’s *t*-test (* *p* < 0.05; ** *p* < 0.001).

We previously identified several herbal extracts with pro- or anti-adipogenic activities in C3H10T1/2 cells [24]. From this screening, we recognized *Chloranthus japonicus* as a possible pro-adipogenic herbal product. To verify the effects of *Chloranthus japonicus*, the methanol extract was used to treat C3H10T1/2 cells and differentiated them to adipocytes. The extracts dramatically increased lipid accumulation in the C3H10T1/2 cells (Figure 1A). The expression of adipogenic markers, *Pparγ, Fabp4, Cd36, C/EBPα*, adiponectin, and *Lpl* increased after 2–6 days of treatment with *Chloranthus japonicus* methanol extract indicating the stimulatory effects of *Chloranthus japonicus* on adipogenic differentiation in C3H10T1/2 cells (Figure 1B). Consistently, the methanol extract promoted the adipocyte differentiation of preadipocyte 3T3-L1 cells (Figure 1C,D).

### 3.2. Chloranthus Japonicus Hexane Fraction Is the Most Potent Stimulator of Adipogenesis in C3H10T1/2 and 3T3-L1 Cells 

**Figure 2:**
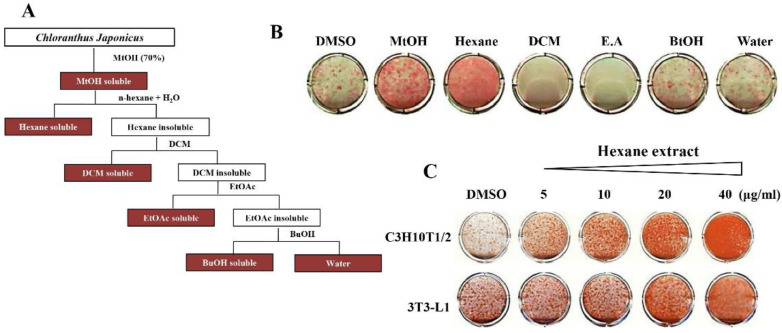
*Chloranthus japonicus* hexane extract promotes adipogenesis. (**A**) Preparation of fractionated extracts from *Chloranthus Japonicus*. *Chloranthus Japonicus* was successively extracted in methanol, n-hexane, dichloromethane (DCM), ethyl acetate (E.A), butanol (BtOH), and water. (**B**) The adipogenic effects of various solvent-fractionated extracts in C3H10T1/2 cells. (**C**) The adipogenic effects of *Chloranthus japonicus* hexane extract (CJHE) in C3H10T1/2 (upper panel) and 3T3-L1 (lower panel) cells.

**Figure 3:**
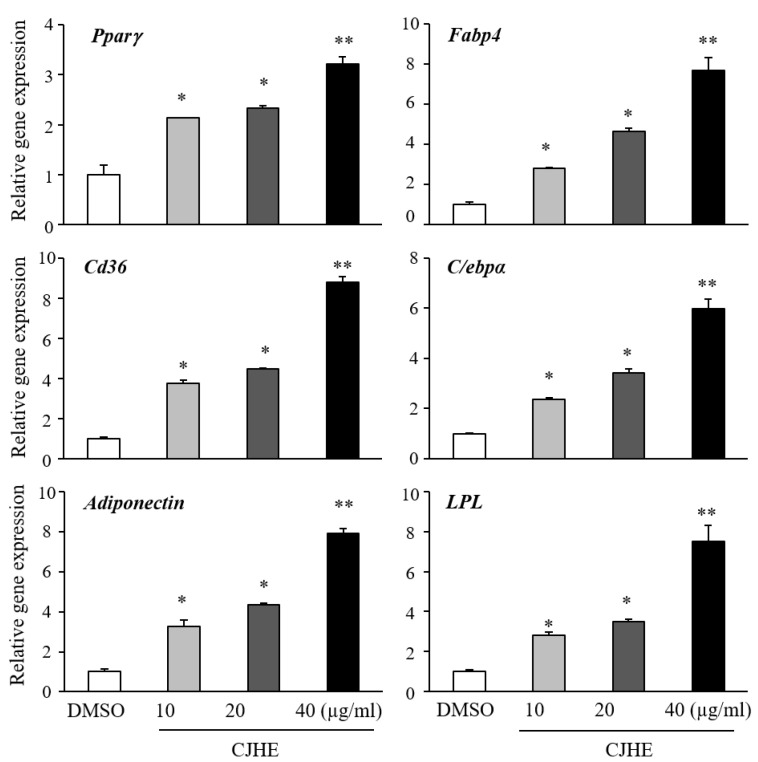
*Chloranthus japonicus* hexane extract increases the expression of adipocyte markers. C3H10T1/2 cells were differentiated in the presence of *Chloranthus japonicus* hexane extract at the indicated doses for 4 days. Expression of adipocyte markers was measured by real-time PCR. The data represent the mean ± standard error of the mean (SEM) of triplicates. Statistical significance was determined relative to the controls by the Student’s *t*-test (* *p* < 0.05; ** *p* < 0.001).

We further separated the *Chloranthus japonicus* extracts into hexane, dichloromethane (DCM), ethyl acetate (E.A), n-butanol, and Water (H_2_O) to enrich the ingredients exhibiting the pro-adipogenic effects of *Chloranthus japonicus* (Figure 2A). The *Chloranthus japonicus* hexane fraction (CJHE) was the most potent promoter of lipid accumulation in C3H10T1/2 adipogenic differentiation (Figure 2B). The hexane fractions similarly promoted the differentiation of 3T3-L1 cells (Figure 2C). Consistently, the expression levels of adipocyte markers was increased by the treatment with CJHE in C3H10T1/2 cells (Figure 3). 

### 3.3. The Pro-Adipogenic Effect of CHJE Is Dependent upon Dexamethasone and Insulin 

**Figure 4:**
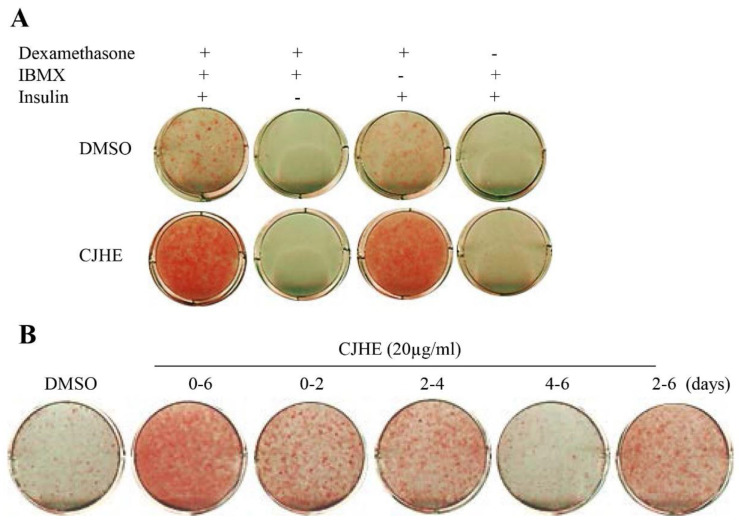
Pro-adipogenic effects of CJHE in 3T3-L1 cells. (**A**) Pro-adipogenic actions of CJHE in various adipogenesis-stimulating conditions. 3T3-L1 cells were differentiated in media containing DMI, DM, DI, or MI and treated with CJHE (20 µg/mL) for 6 days. (**B**) Pro-adipogenic CJHE primarily acts during the early stages of adipocyte differentiation. 3T3-L1 cells were treated with CJHE from 0 to 2, 2 to 4, 4 to 6, 0 to 2, and 0 to 6 days. The differentiated cells were stained with Oil Red O.

To further investigate the effects of CJHE on adipogenesis, we treated CJHE in various adipogenesis-stimulating conditions. 3T3-L1 cells were stimulated into adipocytes in media supplemented with dexamethasone, isobutyl-1-methylxanthine, and insulin. Treatment with CJHE in either DI or DMI-supplemented conditions but not others increased the capacity for lipid accumulation compared to the control (Figure 4A). These data indicate that both dexamethasone and insulin were essential but isobutyl-1-methylxanthine was not required for the promotion of adipogenesis. 

### 3.4. The Pro-Adipogenic Effect of CHJE Is Critical during the Early Adiogenic Stages 

We assessed the temporal effects of CJHE in adipocyte differentiation. To investigate the temporal effects of CJHE, 20 µg/mL of CJHE were treated at 0–2, 2–4, 4–6, 2–6, and 0–6 days of adipocyte differentiation. Treatments with CJHE at 0–2, 2–4, 2–6, and 0–6 days promoted adipocyte differentiation of 3T3-L1 cells. However, treatment from 4 to 6 days did not stimulate adipocyte differentiation, with levels similar to degrees in control (DMSO)-treated cells (Figure 4B). These results show that CJHE acted at the early stage (days 0–4) to promote adipogenesis. 

### 3.5. CJHE Inhibits the Wnt Pathway 

**Figure 5:**
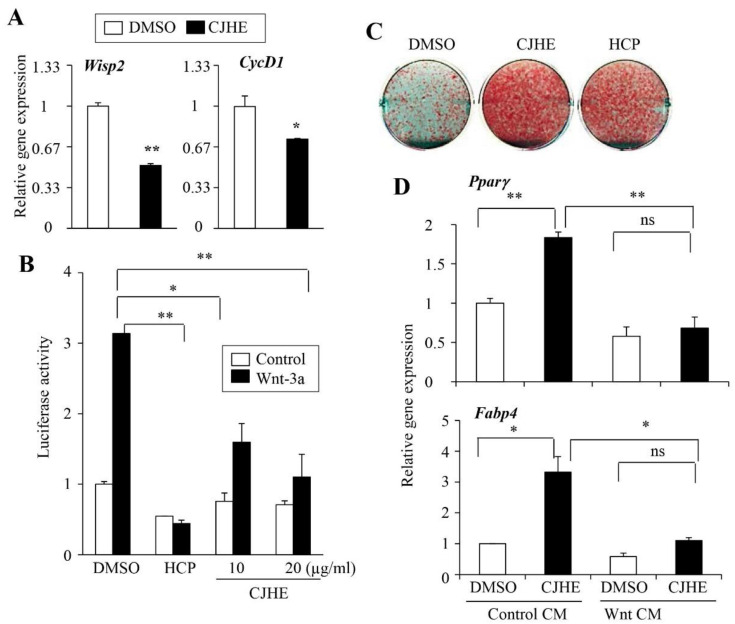
CJHE inhibits Wnt signaling in 3T3-L1 cells. (**A**) 3T3-L1 cells were treated with CJHE for 24 h and the level of Wnt target genes, *Wisp2* and cyclin D1 (*CycD1*) was assessed. (**B**) CJHE inhibits Wnt reporter activity. 293FT cells were transfected with Wnt (TOP-Luc) reporter and treated with bovine serum albumin (BSA), control, or purified Wnt-3a, followed by treatment with 10 or 20 µg of CJHE or a known Wnt chemical inhibitor, hexachlorophene (HCP, 5 µM) for 24 h. Statistical significance was determined by the Student’s *t*-test (* *p* < 0.05). (**C**) HCP, a Wnt chemical inhibitor stimulated adipogenesis. 3T3-L1 cells were differentiated in the presence of CJHE or HCP for 6 days and lipid accumulation was determined by Oil Red O. (**D**) 3T3-L1 cells were differentiated in the presence of control-conditioned media or Wnt-conditioned media and treated with CJHE or HCP for 6 days. Gene expression was assessed by real-time PCR. The data represent the mean ± standard error of the mean (SEM) of triplicates. Statistical significance was determined by the Student’s *t*-test (* *p* < 0.05; ** *p* < 0.001).

Previous studies showed that various phytochemicals including shizukaol D and F found in *Chloranthus japonicus* were bioactive compounds. Shizukaol D was shown to suppress the Wnt signaling pathway [25], an important physiological modulator of Pparγ expression and adipogenesis [4,26]. Based on these reports, we reasoned that CJHE may act on the Wnt signaling pathway to stimulate Pparγ expression and adipogenesis. To test this possibility, we treated 3T3-L1 cells with CJHE (at 20 µg/mL) for 12 hours and measured the expression of the previously known Wnt target genes, *Wisp2* and cyclinD1 (*CycD1*). We observed the reduced expression of *Wisp2* and *CycD1* in 3T3-L1 cells (Figure 5A). We also found similar inhibitory effects for the expression of *Wisp2* and *CycD1* in CJHE-treated C3H10T1/2 cells (Figure S1). 

To further examine the activities of CJHE on the Wnt signaling, we employed a Wnt-pathway selective reporter. We transiently transfected a Wnt-specific luciferase reporter plasmid, TOP flash, into 3T3-L1 cells and treated with CJHE. We observed that purified Wnt3a protein-driven luciferase TOP flash was inhibited by treatment with CJHE similar to the inhibitory effects of the known Wnt inhibitor, HCP (Figure 5B). 

To further confirm the pro-adipogenic effects of Wnt inhibition in our system, we treated cells with HCP, a known Wnt inhibitor, and induced adipogenesis. Consistent with the stimulatory effects of Wnt inhibition on adipogenesis, we verified the increased adipocyte differentiation by HCP and CJHE (Figure 5C). Conversely, Wnt3a-conditioned media collected as previously described [23], suppressed the effects of CJHE on *Pparγ* and *Fabp4* expression further, confirming the critical role of Wnt signaling in CJHE-mediated adipogenesis (Figure 5D). Similarly, the increased lipid accumulation by CJHE was partly suppressed in the presence of Wnt3a protein (Figure S2). 

### 3.6. Wnt Signaling Is Essemtial for the Effects of CJHE 

**Figure 6:**
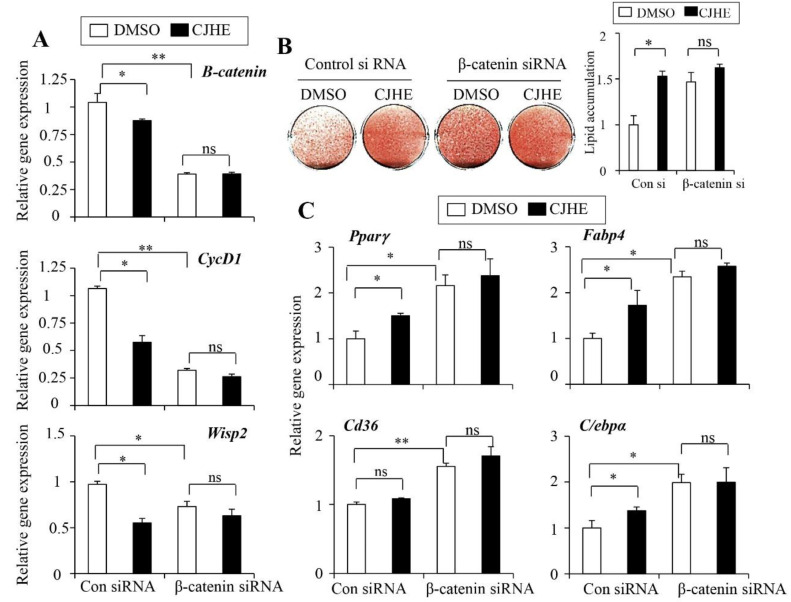
CJHE promotes adipocyte differentiation through the Wnt/β-catenin signaling. (**A**) Knockdown of β-catenin expression in 3T3-L1 cells attenuated the CJHE inhibitory effects on the Wnt target genes. The expression of β-catenin, *Wisp2,* and *CycD1* in the control and β-catenin-targeting siRNA-transfected cells was measured. (**B**) Knockdown of β-catenin expression blunted the stimulatory effects of CJHE on lipid accumulation (left). Lipid accumulation was quantified (right). Control and β-catenin siRNA-transfected 3T3-L1 cells were treated with DMSO or CJHE (20 μg/mL) and differentiated for 6 days. (**C**) Silencing β-catenin blunted the inhibitory effect of CJHE on the expression of adipocyte markers. The data represent the mean ± standard error of the mean (SEM) of triplicates. Statistical significance was determined by the Student’s *t*-test (* *p* < 0.05; ** *p* < 0.001).

To show that Wnt signaling was critical for the pro-adipogenic effect of CJHE, we used CJHE to treat β-catenin-abrogated 3T3-L1 cells using siRNA. We employed siRNA verified to effectively silence β-catenin expression [27]. Control or β-catenin siRNA-transfected 3T3-L1 cells were differentiated and treated with CJHE. The expression of β-catenin and its target genes *Cycd1* and *Wisp2* was successfully decreased in the β-catenin siRNA transfected cells. We found consistent decreases in *Cycd1* and *Wisp2* expression in control siRNA-transfected cells by CJHE treatment. However, the effects on the expression of *Cycd1* and *Wisp2* were impaired in the β-catenin-targeted siRNA-transfected 3T3-L1 cells (Figure 6A). We further assessed the effects of CJHE in adipogenesis of β-catenin-deficient cells. As expected, β-catenin-deficient cells showed increased lipid staining and adipogenic markers expression (Figure 6B,C). The increase in adipogenic potential by CJHE treatment was not further induced in the β-catenin siRNA-transfected cells (Figure 6B). Similarly, the expression of *Pparγ*, *Fabp4*, *Cd36*, and *C/EBPα* was increased in control CJHE treated cells. However, the stimulatory action of CJHE on these adipocyte markers was significantly blunted in β-catenin-defective 3T3-L1 cells (Figure 6C). Together, these data demonstrated that although the exact molecular actions still need further investigation, Wnt/β-catenin signaling, at least in part, was necessary for the effect of CJHE on adipocyte differentiation. 

### 3.7. Differential Effects of the Extracts Generated from Different Plant Parts on Adipogenesis

**Figure 7:**
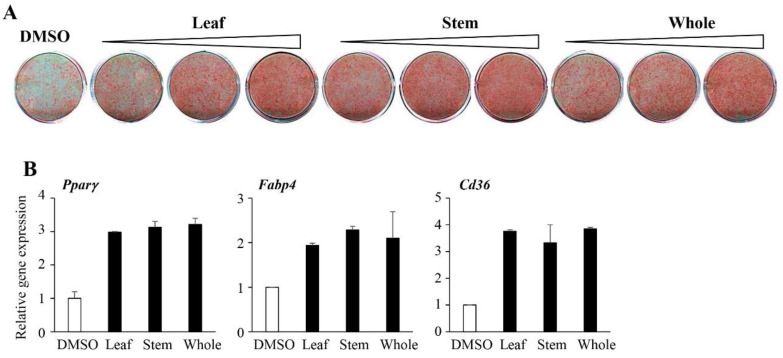
The pro-adipogenic activities of CJHE extracts from different plant parts. (**A**) 3T3-L1 cells were treated with extracts from leaves, stems, or the whole plant (at 10, 20, and 40 µg/mL), differentiated for 6 days, and assessed by Oil Red O. (**B**) The expression of adipocyte markers from the differentiated 3T3-L1 cells treated with 40 µg/mL of leaf, stem, or whole plant extracts was quantified. The data represent the mean ± standard error of the mean (SEM) of triplicates. Statistical significance was determined by the Student’s *t*-test (* *p* < 0.05; ** *p* < 0.001).

It has been shown that plants collected in different seasons and different parts of the plant could display different activities [28,29]. Thus, it is possible that different parts of *Chloranthus japonicus* may also exhibit differential effects on adipogenic activities. To investigate this possibility, we analyzed the effects of extracts originating from the leaves or stems and compared them to the aerial parts of the plant (whole plants). Extracts from the leaves, stems, and whole plants promoted lipid accumulation that was comparable to the effects of the extracts from the whole plants (Figure 7A). Consistently, *Pparγ*, *Fabp4*, and *Cd36* expression in cells treated with the leaf or stem extracts mirrored the effects of the extracts from the whole plants (Figure 7B). These data show that the stems and leaves of *Chloranthus japonicus* may act similarly to promote lipid accumulation and adipocyte differentiation.

## 4. Discussion

Pparγ, when stimulated by TZDs, a class of Pparγ ligands, controls glucose homeostasis in diabetes [8]. However, TZDs have been shown to cause weight gain and increase the risk of congestive heart failure, suggesting the need for alternatives to treat type 2 diabetes [7]. Recent evidence shows that the modulation of Pparγ phosphorylation events (S273) protect against insulin resistance [11,12], indicating that the selective regulation of Pparγ can provide new strategies against metabolic diseases, including diabetes, without deleterious effects [13]. Given the regulatory roles of Pparγ in diabetes [6], the identification of Pparγ inducers from herbal products and the further isolation of bioactive compounds can be useful for treating insulin resistance [14]. Previous studies reported that *Chloranthus japonicus*, primarily distributed throughout northern China, Korea, and Japan, could improve conditions such as injuries, rheumatic arthralgia, bone fractures, pulmonary tuberculosis, neurasthenia, and dysregulated glucose metabolism [17,18,30]. However, its activity on adipogenesis has not been reported. In this study, we investigated the effects of *Chloranthus japonicus* in adipocyte differentiation and Pparγ expression. We also identified the hexane fraction of *Chloranthus japonicus* as a prominent inducer of adipogenesis and elucidated its underlying molecular mechanisms. 

Natural products are well-acknowledged sources of small molecules biologically active in various signaling pathways and these are often used for drug development [31,32]. Similarly, various plant extracts exert biological activities by acting on the Wnt signaling pathway [33,34]. For example, *Euodia sutchuenensis* stimulates the Wnt pathway to increase osteoblast differentiation [35]. Furthermore, numerous Wnt regulators including indirubins [36,37], andrographolide [38], flavone fukugetin [39], curcumin [40], and harmine [14] have been discovered from natural products. In particular, harmine found in *Peganum harmala* and *Banisteriopsis caapi* induces Pparγ expression by inhibiting the Wnt signaling pathway [14,23]. These findings suggest that various herbal products and phytochemicals mediate biological activity by acting on the Wnt pathway. Other phytochemicals such as delphinidin [41], coumestrol [42], and epigallocatechin gallate [43] regulate both adipogenesis and Wnt signaling. Here, we also explored the links between *Chloranthus japonicus*, Pparγ, and the Wnt signaling pathway. We showed that *Chloranthus japonicus* suppressed Wnt-downstream target gene expression. The treatment of 3T3-L1 cells with Wnt-conditioned medium or purified Wnt-3a suppressed the pro-adipogenic effects of *Chloranthus japonicus*. We also showed that treatment with a chemical inhibitor of Wnt-signaling mimicked the effects of *Chloranthus japonicus* in adipocyte differentiation and that siRNA-mediated knockdown of β-catenin abolished the ability of *Chloranthus japonicus* to stimulate differentiation. These data suggest that *Chloranthus japonicus* acted on the Wnt signaling pathway to regulate PPARγ during adipogenesis. However, we cannot entirely exclude the possibility that CJHE may act on other signaling pathways to regulate PPARγ expression. 

We showed that CJHE promoted lipid accumulation in C3H10T1/2 and 3T3-L1 cells. The extracts also increased the expression of *Pparγ* mRNA and its direct target genes. In this study, we did not identify the bioactive compounds in CJHE responsible for the observed activities. As polyphenols were highly prevalent in herbal extracts including *Chloranthus japonicus*, these might be bioactive pro-adipogenic compounds [16,31]. Indeed, sesquiterpene and diterpenoids such as chlojaponilactone B, shizukaol D, and shizukaol F have been identified [16,19,20,25]. Interestingly, selected sesquiterpenes, bilobalide, dehydroleucodine, and zaluzanin C were shown to regulate adipogenesis [44,45,46], suggesting the possibility that these bioactive compounds are likely to confer the pro-adipogenic effects of the CJHE. Further analysis is necessary to identify the major bioactive compounds for stimulating adipogenesis. In the current study, we investigated the effect of CJHE and molecular mechanism during adipocyte differentiation. Therefore, future studies on the effects of CJHE in the PPARγ expression and insulin sensitivity in the differentiated adipocytes will be also critical to determine utility of CJHE for insulin resistance and diabetes.

## Data Availability

Not applicable.

## Supplementary Materials

The following are available online at https://www.mdpi.com/2075-1729/11/3/241/s1, Figure S1: CJHE inhibits Wnt target gene expression in C3H10T1/2 cells, Figure S2: Wnt3a inhibits the effects of CJHE on adipocyte differentiation.
