# Supplementary material for: Hexane Extract of Chloranthus japonicus Increases Adipocyte Differentiation by Acting on Wnt/β-Catenin Signaling Pathway
Source: Life (Basel). 2021 Mar 15;11(3):241. doi: 10.3390/life11030241 (PMC7999792; doi:10.3390/life11030241)
Supplement: Supplementary file 1 [file life-11-00241-s001.pdf]

life

Supplementary Materials

Hexane Extract of *Chloranthus japonicus* Increases Adipocyte Differentiation by Acting on Wnt/β-catenin Signaling Pathway

Ui Jeong Yun [1], Chu Won Nho [2], Kye Won Park [1,*] and Dong Kwon Yang [3,*]

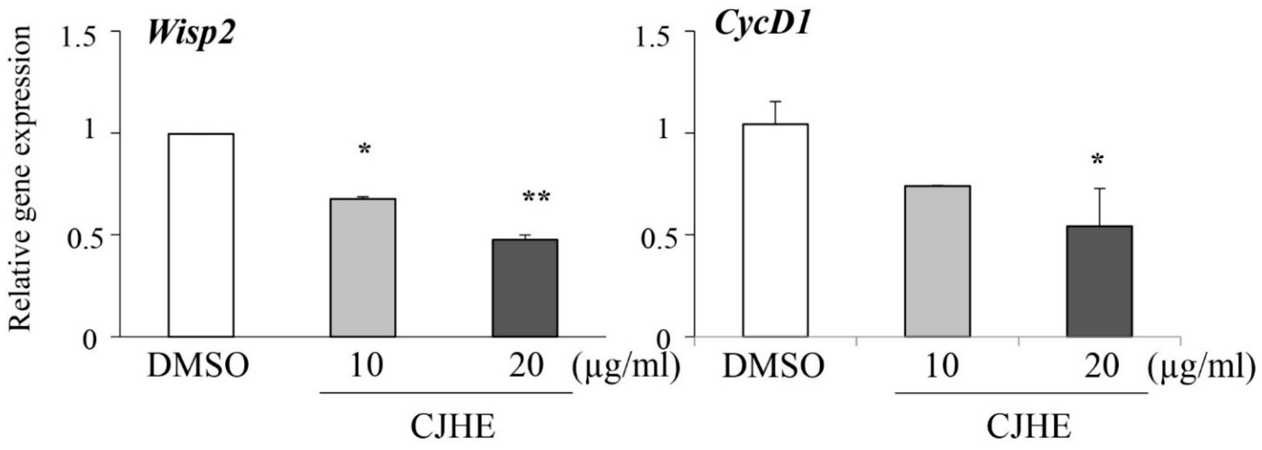

Figure S1. CJHE inhibits Wnt target gene expression in C3H10T1/2 cells. C3H10T1/2 cells were treated with CJHE for 24 h and the expression of Wnt target genes *Wisp2* and *CycD1* was assessed by real-time PCR. The data shown represent the mean ± standard error of the mean (SEM). Statistical significance was determined relative to the controls by the Student's t-test (*$p < 0.05$; **$p < 0.001$).

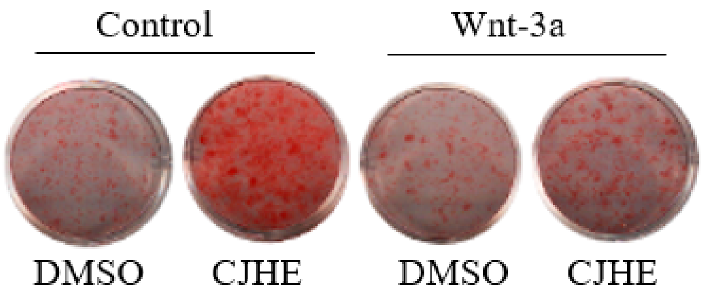

Figure S2. Wnt3a inhibits the effects of CJHE on adipocyte differentiation. 3T3-L1 cells were differentiated into adipocytes in the presence of BSA, control, or purified Wnt-3a and treated with 20 μg of CJHE for 6 days. Lipid accumulation was assessed by Oil Red O staining.
